# Effect of daratumumab on stem cell yields in patients with newly diagnosed multiple myeloma: a report from the Multiple Myeloma Group

**DOI:** 10.1038/s41409-024-02260-z

**Published:** 2024-03-09

**Authors:** F. Fazio, M. Passucci, J. Micozzi, F. Di Landro, L. Fianchi, T. Za, V. M. Manieri, O. Annibali, L. Cupelli, V. Bongarzoni, S. Gentili, L. De Padua, E. Crisanti, M. G. Garzia, A. Rago, A. Piciocchi, A. Mengarelli, S. Morè, V. De Stefano, M. S. Bafti, M. Martelli, M. T. Petrucci

**Affiliations:** 1 Hematology, Azienda Policlinico Umberto I—Department of Translational and Precision Medicine, Sapienza University of Rome, Rome, Italy; 2 Section of Hematology, Department of Radiological and Hematological Sciences, Catholic University, Fondazione Policlinico Universitario A. Gemelli Istituto di Ricovero e Cura a Carattere Scientifico (IRCCS), Rome, Italy; 3 Clinica di Ematologia Azienda Ospedaliero-Universitaria delle Marche, Ancona, Italy; 4 Unit of Hematology, Stem Cell Transplantation, University Campus Bio-Medico, Rome, Italy; 5 UOC Hematology, Hospital S. Eugenio, Rome, Italy; 6 Department of Hematology San Giovanni-Addolorata Hospital, Rome, Italy; 7 UOSD Ematologia Civitanova Marche AST Macerata, Rome, Italy; 8 Hematology Unit, Fabrizio Spaziani Hospital, Frosinone, Italy; 9 Hematology and Transplant Unit, Santa Maria Goretti Hospital, AUSL, Latina, Italy; 10 Department of Hematology, Hematology San Camillo Forlanini Hospital Rome Italy, Rome, Italy; 11 UOSD Ematologia ASL Roma1, Rome, Italy; 12 Italian Group for Adult Hematologic Diseases (GIMEMA) Data Center, Rome, Italy; 13 Hematology Unit, IRCCS Regina Elena National Cancer Institute, Rome, Italy; 14 Department of Immuno-Hematology and Transfusional Medicine, Policlinico Umberto I, Rome, Italy

**Keywords:** Myeloma, Diseases

## To the Editor:

High dose chemotherapy followed by autologous stem cell transplantation (ASCT) has been considered the standard of therapy for younger fit patients with newly diagnosed multiple myeloma (MM), since several randomized trials demonstrated a survival benefit for ASCT compared to conventional chemotherapy, even in the era of novel induction triplet and quadruplet therapy regimens [1–3].

A minimum of 2 × 10^6^ CD34+ cells/Kg need to be collected and reinfused after a high dose of melphalan to ensure an adequate hematopoietic reconstitution after ASCT. Furthermore, a significant proportion of multiple myeloma patients could be subjected to tandem ASCT at first line therapy or salvage ASCT at first relapse, so the optimal goal is to collect at least 4 × 10^6^ CD34+ cells/Kg [4]. The chemokine receptor antagonist plerixafor is commonly used on demand as a rescue for patients with poor mobilization [5].

Daratumumab is a human IgG monoclonal antibody targeting CD38 on clonal plasma cells with a direct on-tumor and immunomodulatory mechanisms of action [6].

Clinical efficacy and safety of daratumumab-based combination induction therapy for transplant eligible newly diagnosed MM patients was firstly investigated in the phase II Griffin trial [7]. In the phase III CASSIOPEIA study, daratumumab plus bortezomib, thalidomide and dexamethasone (D-VTd) showed a significantly improved progression free-survival (PFS) and MRD-negativity rate compared to VTd and, currently, D-VTd represents the standard of care in Europe for newly diagnosed transplant eligible MM patients [8]. Daratumumab exposure it was associated with a lower median stem cell yield and more frequent plerixafor use without a significant impact on hematopoietic stem cell reconstitution after ASCT [9].

Daratumumab targets also on CD34+ hematopoietic progenitor cells and it is known that mobilized CD34+ cells are critical for ASCT. Daratumumab could be involved in CD38 expression on CD34+ cells, possible to affect mobilization kinetics and lineage-specific progenitor proliferative capacity. Considering the role of CD38 in promoting the leukocytes motility during inflammation, it is possible that daratumumab could interfere with CD34+ stem cells diapedesis through the vassal endothelium of bone marrow microenvironment, blocking their passage in peripheral blood after mobilization signal [10].

The aim of this study is to investigate the possible impact of daratumumab-based induction therapy on first attempt peripheral blood stem cell collections, and to identify possible features that could affect stem cell yields.

We retrospectively evaluated 78 consecutive patients with NDMM managed at 12 Italian Hematology Centers who received daratumumab-based quadruplet induction regimens (D-VTd), followed by stem cell mobilization therapy, between November 2021 and March 2023. Following induction, patients underwent mobilization therapy as per institutional guidelines. Per protocol, sufficient stem cells should be harvested to enable at least one ASCT, in accordance with institutional standards. The baseline clinical characteristics and the frontline induction therapy were summarized in Table 1. The median age of the entire cohort was 61 years (IQR 56–66) and, 48 patients were male (62%). Most patients were ISS I (40%) and R-ISS II (43%). Out of 65 patients evaluable for cytogenetic abnormalities, 34 (52%) had a high-risk cytogenetic abnormality, defined as del17(p); t(4;14), t(14;16) or ampl(1q), and 31 patients had standard risk cytogenetics. Considering CRAB criteria, bone lytic lesions were the most common myeloma defining events (90%), followed by anemia (45%), hypercalcemia (17%) and renal insufficiency (15%). The median bone marrow plasma cells was 60% (IQR 35–75) and 9 patients (12%) had a baseline plasmacytoma. The majority of patients received the full dose of thalidomide as per protocol (100 mg/die) and 92% of patients underwent to 4 cycles of induction therapy before stem cell mobilization chemotherapy. Forty-one patients (54%) experienced non-hematologic adverse events during induction therapy, mainly peripheral neuropathy. For this reason, thalidomide and bortezomib dose was reduced in 26% and 15% of patients, respectively. Globally, 13% of patients showed hematologic adverse events, in most cases thrombocytopenia and leucopenia. After induction therapy, the overall response rate was 100% and 66% of patients achieved a very good partial response or better.

**Table 1:** Baseline clinical characteristics and study results.

Characteristcs	Pts (*N* = 78)	%
Gender		
Male	48	62
Female	30	38
Age (years)		
Median (range)	61 (56–66)	
ISS		
I	31	40
II	27	35
III	19	24
Not available	1	1
R-ISS		
I	22	28
II	29	37
III	17	22
Not available	10	13
Bone marrow plasma cell infiltration		
Median (range)	60 (35–75)	
Cytogenetic risk, n(%)		
High risk	34	43
Standar risk	31	40
Missing/not available	13	17
Mobilization regimen, n(%)		
G-CSF	3	3.8
CTX + G-CSF	70	90
G-CSF + PLERIXAFOR	1	1.3
CTX + G-CSF + PLERIXAFOR	4	5.1
Number of days of CTX, n(%)		
1	42	55
2	34	45
Number of plerixafor dose, n(%)		
1	17	71
2	7	29
Total dose of CTX (gr) Median (range)	3.2 (3.6–5)	
Response after induction therapy		
sCR	10	13
CR	16	21
VGPR	40	51
PR	12	15
CD34+ stem cell collection Median (range)	7.6 (5.9–9.9)	
Time between last day of induction and the first day of mobilization therapy Median (range)	31 (21–45)	
Plerixafor on demand, n(%)	24	30
Bone marrow function pre-mobilization therapy, median(range)		
Hb, g/dL	12.7 (12.1–13.7)	
Platelets, mm^3^	234 (185–310)	
Neutrophils, mm^3^	3000 (2120–4178)	

Analyzing the possible impact of the inclusion of daratumumab into induction therapy, we observed that, globally, 73/78 patients (93%) met the collection goal after mobilization therapy. Nevertheless, 5 out of 73 patients (7%) and 2 out of 73 patients (3%) required a second and third mobilization attempt, respectively. Moreover, 5 out of 78 patients (6%) failed peripheral stem cell collection.

Mobilization of peripheral blood cells was induced with a combination of cyclophosphamide (CTX) with granulocyte colony stimulating factor (G-CSF) in 70 patients (90%). Three patients (4%) received G-CSF alone; one patient (1%) received G-CSF plus plerixafor, and 4 patients (5%) received CTX plus G-CSF and plerixafor. The median dose of CTX administered was 4.2 gr (IQR 3.6–5), and the majority of patients (55%) received a full dose of CTX on one day. However, 34 out of 78 patients (43%) received split dose of CTX over two days. The median daily dose of G-CSF was 735 mcg (IQR 600–960). The median number of CD34+ stem cells collection yield for the entire cohort was 7.6 × 10^6^ cells/Kg (IQR 5.9–9.9). Furthermore, the median time between the last day of induction therapy and the first day of mobilization therapy was 31 days (IQR 21–45). Overall, plerixafor on demand was administered in 24/78 pts (30%) failing to achieve the desired collection goals, confirming that a higher use of plerixafor was necessary in the daratumumab-based induction therapy, compared to our previously experience based on VTd induction therapy, suggesting an additional health spending for stem cell collection. Besides the use of daratumumab in induction therapy, univariate analysis showed that a baseline plasmacytoma was associated with a lower rate of collection goal, after the first mobilization attempt (*p* = 0.028), confirmed in multivariate logistic regression model too (*p* = 0.02). In our experience, gender, number of induction cycles, depth of response at the time of apheresis and type of mobilization therapy had no significant impact on peripheral stem cell yield. Patients with a median lower pre-mobilization therapy level of neutrophils showed a significantly lower collection goal after the first mobilization attempt (*p* = 0.021), possibly due to prolonged hematological toxicity after induction therapy.

Over the last decade, the introduction of several novel agents, such as immunomodulatory drugs (IMiDs), proteasome inhibitors (PIs) and monoclonal antibodies (moAbs) for the treatment of NDMM has improved the outcome of MM patients, in terms of PFS and overall survival [11]. However, high-dose chemotherapy followed by ASCT remains a standard of care for younger fit patients. Currently, EHA-ESMO guidelines recommend tandem ASCT as a therapeutic option for patients with high-risk features and those who do not achieve at least a VGPR after the first ASCT [12]. Furthermore, a second ASCT should be also considered as an option at the time of relapse in selected patients.

As previously reported in the CASSIOPEIA trial, we confirmed that the median number of CD34+ stem cells collected after chemo-mobilization therapy was lower for patients receiving D-VTd, compared to patients treated with VTd as frontline induction therapy.

In addition, we showed that, the addiction of daratumumab to the frontline induction treatment, is associated with a higher use of plerixafor on demand to meet the collection goal at the first mobilization attempt, with an increase in health spending.

A larger cohort of patients are warranted to understand the exact mechanism by daratumumab should interferes with CD34+ stem cells yield.

A new mobilization therapy schedule is necessary to guarantee an adequate stem cell yield in order to perform a safety ASCT.
